# PANDAS/PANS in the COVID-19 Age: Autoimmunity and Epstein–Barr Virus Reactivation as Trigger Agents?

**DOI:** 10.3390/children10040648

**Published:** 2023-03-30

**Authors:** Stefano Pallanti, Michele Di Ponzio

**Affiliations:** 1 Department of Psychiatry and Health Sciences, Institute of Neurosciences, 50121 Florence, Italy; 2 Department of Psychiatry and Behavioural Sciences, Albert Einstein College of Medicine, New York, NY 10461, USA; 3 Department of Psychology and Cognitive Studies, Institute of Neurosciences, 50121 Florence, Italy

**Keywords:** PANDAS, PANS, COVID-19, neuroinflammation, immunology

## Abstract

COVID-19 impacted the entire world’s population, frequently resulting in long-lasting neuropsychiatric complications. Furthermore, social distancing, lockdowns and fear for one’s personal health worsen individual psychological wellbeing, especially in children and adolescents. Herein, we discuss the results of studies that specifically reported data about the impact of the COVID-19 pandemic or infection on children with Pediatric Acute-Onset Neuropsychiatric Disorders (PANS). Furthermore, we present the cases of five adolescents with PANS whose symptomatology increased following SARS-CoV-2 infection. What emerged from this study was that COVID-19 resulted in the exacerbation of obsessions, tics, anxiety and mood symptoms and decreased wellbeing. Moreover, new symptoms, as well as new PANS cases, are reported to have arisen after COVID-19 infection. Here, we hypothesize that the pathogenic mechanisms of silent viruses, such as the Epstein–Barr virus, are related to neuroinflammation, immune responses and reactivation, with additional roles played by social-isolation-related inflammatory processes. The discussion of PANS, which represents a model of immune-mediated neuropsychiatric manifestations, is particularly relevant, with the aim of uncovering the mechanisms that lead to neuropsychiatric Post-Acute COVID-19 Syndrome (PACS). Prospects for future studies and treatment implications are discussed.

## 1. Introduction

At the time that this review was written, more than 600 million people had been infected with the SARS-CoV-2 virus. The effects of COVID-19 spread through the entire human body [1], including the brain, resulting in neurological and psychiatric symptoms [2,3]. In a large multicentric international study, 80% of hospitalized COVID survivors reported neurologic symptoms [4]. Among these neurological and neuropsychiatric abnormalities, the most common are headache, confusion, memory difficulties, anosmia, hypogeusia, anxiety symptoms, Guillain–Barre syndrome, encephalitis, seizures and cerebrovascular stroke [5,6,7].

Furthermore, neuropsychiatric symptoms persist after infection and, in some cases, appear weeks later. A total of 37% of post-COVID patients experiences fatigue, 33% experience brain fog, and around 20–30% experience anxiety or depressive symptoms [8]. This condition has been named Neuropsychiatric Post-Acute COVID-19 Syndrome (PANS) [9].

The effects of respiratory infections on psychiatric disorders are well-known. Indeed, as seen in Severe Acute Respiratory Syndrome (SARS) survivors, SARS infection is associated with an increased risk of developing a psychiatric morbidity [10]. Indeed, more than 40% of SARS survivors experienced at least one psychiatric disorder in a 4-year follow-up, with the most common types being post-traumatic stress disorders (54.5%), depression (39%), somatoform pain disorder (36.4%), panic disorder (32.5%) and obsessive compulsive disorder (OCD) (15.6%) [10].

COVID-19 impacts not only the lives of infected individuals but also those of the uninfected. Indeed, quarantine and social isolation decreased wellbeing and negatively affected people’s mental health [11,12]. An online survey showed that 60% of respondents reported the onset of OCD symptoms during the pandemic, with an increased likelihood of presenting with generalized anxiety disorder and depression [13]. A large international survey study reported that most OCD patients perceived a worsening of their symptoms during the pandemic [14]. This worsening was associated with an increased demand for psychological and pharmacological therapies. Furthermore, the COVID-19 pandemic particularly impacted the mental health of children and adolescents [15]. Indeed, the psychological state of almost 80% of children was negatively affected by the pandemic and quarantine [16]. Acute COVID-19 infection resulted in long-term clinical consequences in children [17], and the symptomatology of children with comorbid neuropsychiatric disorders was also further exacerbated by COVID-19 infection. Autoimmunity and inflammation are regarded as potential pathogenic mechanisms of the worsening or new onset of symptoms following COVID-19 infection and the pandemic [3,18,19]. Considering the overall impact of COVID-19 on neuropsychiatric symptomatology in children and its potential autoimmunological explanation, Pediatric Autoimmune Neuropsychiatric Disorders (PANS) represent an optimal model of immune-mediated neuropsychiatric manifestations. Therefore, a discussion of the potential relationship between PANS and COVID-19 and its potential mechanism of alteration is needed.

In this review, we discuss the results of studies that specifically reported associations between the COVID-19 pandemic or infection and children with Pediatric Acute-Onset Neuropsychiatric Disorders (PANS), with a focus on the involvement of the Epstein–Barr virus. Furthermore, we report on the cases of five adolescents whose symptomatology was aggravated following SARS-CoV-2 infection. Finally, we aimed to explore the potential implications of the common pathogenic mechanisms.

## 2. COVID-19 Impact on Children and Adolescents

Among children, the pandemic and related-social isolation have been associated with the onset of anxiety–depressive symptoms and stress [20]. Specifically, it was reported that in the first year of the pandemic, one of four youths showed more severe depression symptoms, while one of five youths showed more anxiety symptoms [21]. Moreover, children and adolescents experienced increased inattention and irritability and became more dependent on their caregivers [15,22]. In the clinical population, an exacerbation of the frequency of contamination obsessions and cleaning/washing compulsions was observed in children with OCD, with more than one-third of the subjects showing an increase of at least 30% in their total CY-BOCS [23], as well as increased anxiety and depressive symptoms [24]. The latter result was confirmed by an observational longitudinal study of children and adolescents with psychiatric or neurological disorders, who showed a worsening of anxiety and obsessive symptoms [25]. Risk factors more commonly related to the onset of symptoms during the lockdown have been observed to be related to parental stress, fear of contagion, changes in daily routine and social isolation [22,26,27].

Concerning the neuropsychiatric effects of infection on children, an Italian study showed that 66% of patients continued to have symptoms for at least 2 months after COVID-19 [28]. The most prevalent symptoms were fatigue (16,2%) and lack of concentration (11.8%). A meta-analysis [17] then reported the occurrence of Long COVID in children after 12 weeks post-infection with a prevalence of more than 25%, with mood symptoms and fatigue among the most common symptoms. Savino et al. [29] reported five pediatric cases of the onset of neuropsychiatric symptoms following COVID-19 infection. The immune hypothesis is still considered as a potential cause. Several autoimmune diseases and autoinflammatory conditions, such as pediatric inflammatory multisystem syndrome (PIMS), Kawasaki disease and encephalitis in children [30], develop after SARS-CoV-2 infection. Savino et al. [29] found that in one case, the onset of tics following COVID-19 in a child was associated with increased CRP and ASR and antistreptolysin titers, while another case was associated with increased lymphocytes and red blood cells, supporting the neuroinflammatory hypothesis.

## 3. PANS/PANDAS

In 1998, Swedo [31] reported the first description of 50 cases of children who experienced the abrupt onset of OCD or tic disorder symptoms following Group A Streptococcus infection. Swedo coined the term PANDAS, which denotes Pediatric Autoimmune Neuropsychiatric Disorders Associated with Streptococcal infections. Over time, clinicians observed that many episodes of PANDAS symptoms appeared to be triggered by non-Group-A Streptococcal infections. Consequently, new diagnostic criteria were developed to describe PANS, or Pediatric Acute-Onset Neuropsychiatric Disorders [32]. These new criteria (see Table 1) established other factors beyond streptococcal infections as triggering events, including non-infectious triggers, such as environmental factors and metabolic disorders.

While a specific infectious pathogen (streptococcus) is responsible for PANDAS, different microbes may possibly be implicated in the genesis of PANS, such as H1N1 influenza, Epstein–Barr virus and Borrelia burgdorferi (Lyme) disease [33,34,35,36]. Furthermore, PANS is also presumed to be caused by a variety of disease mechanisms with multiple etiologies, such as neuroinflammatory, toxic, environmental, metabolic or endocrine disorders, via the triggering of autoimmune responses [37,38,39,40].

Concerning the PANS phenotype, in a characterization study conducted by Murphy et al. [41] anxiety, emotional lability and low quality of life emerged as key symptoms of all PANS patients. Other peculiarities instead emerged to be specific to only of some pf the patients, and this led to the definition of three clusters of PANS symptoms. PANS youths with elevated streptococcal antibody titers were more likely to have more severe OCD compared to youths without elevated titers. Cluster 1, operationally defined as “core characteristic PANS symptoms”, included five symptoms that were predominantly consistent with the hallmark symptoms of PANS (e.g., emotional lability, anxiety symptoms, sleep disturbances, deterioration in school and behavioral regression). Cluster 2 was defined as “streptococcal-related symptoms” and included eight symptoms that predominantly consisted of symptoms previously described as being associated with GAS infection (e.g., urinary symptoms, ADHD, handwriting deterioration) [41], as well as sensory problems and simple tics. Cluster 3 predominantly included symptoms such as food restriction, mydriasis, fatigue, gastrointestinal problems and depressive symptoms, which are all cytokine-related behavioral symptoms [42]. Therefore, cluster 3 was operationally defined as “cytokine-driven/physiological symptoms.” Moreover, patients with symptoms in cluster 3 exhibited elevated mycoplasma, hallucinations and/or psychotic symptoms, as well as complex tics. 

### Pathogenic Mechanism of PANS/PANDAS

The OCD and tic symptoms of PANDAS are hypothesized to result from either an autoimmune or inflammatory disruption of cortico-striato-thalamo-cortical circuits. Studies have shown structural and functional neuroimaging abnormalities in the basal ganglia [43]. Antibodies isolated from children with PANDAS, compared with control subjects, selectively recognize a subtype of neuron in the basal ganglia of postmortem human brains [44]. A thorough analysis of the literature revealed elevated cytokines (TNF-alpha, IL-6) in OCD [45] and PANS patients [46]. Moreover, the volume of the basal ganglia has been shown to be increased in PANDAS patients compared to controls, and increased activation of the microglia, the brain’s resident immune cells, was observed in PANDAS subjects who were compared to those with Tourette’s syndrome [43]. Moreover, further evidence for the autoimmunity of PANDAS was provided by the discovery of serum and CSF reactions to postmortem human caudate and putamen tissues [43]. 

Family histories of PANS patients revealed a high incidence of autoimmune disorders (80%) [47]. PANS patients also frequently demonstrate coexisting autoimmune and/or inflammatory diseases, most commonly inflammatory back pain (21%) and reactive or persistent arthritis (28%) [48].

## 4. PANS and COVID-19

As stated above, PANS refers to the acute presentation of neuropsychiatric syndrome in children, including OCD. OCD symptoms in adults and children have worsened as an effect of the novel coronavirus (COVID-19) pandemic, as have anxiety and depression symptoms [14]. However, few studies have investigated the possible relationships between PANS/PANDAS and COVID-19 (see Table 2). The relationship between PANS and COVID-19 should be addressed for at least two reasons. First, considering the impacts of the pandemic and direct infection on the mental health of children, as previously reported, children with PANS may have also been considerably affected by this virus. Therefore, this is a first clinical reason. The second is more mechanistic. In fact, in light of the autoimmunity hypothesis of neuropsychiatric complications following COVID-19 infection and the autoimmune origin of PANS, PANS represents a special case to be studied, since it is a model of immune-mediated neuropsychiatric manifestations which could help, in this case, to uncover the mysteries of neuropsychiatric PACS complications.

### 4.1. New Onset or Exacerbation of Symptoms

In two survey studies [49,50], researchers asked caregivers of children and adolescents with PANS to complete standardized questionnaires related to the severity of their children’s symptoms. In both cases, the majority reported that the pandemic negatively impacted their children’s relationships with others, academic performance and wellbeing. These findings are consistent with other parental reports of reduced psychological well-being in children during the pandemic [53]. Patients with PANS/PANDAS syndrome showed an increase in symptoms during the lockdown in 71% of the sample, and the onset of new symptoms was observed in almost one third of the sample [50], being correlated with the presence of sleep disturbances, anxiety and the effects of pandemic stress, such as fear of contracting the virus. Interestingly, 29% of the children showed the onset of new symptoms such as irritability, fears and generalized anxiety [50].

O’Dor and colleagues [49] also reported changes in symptoms in a subgroup of patients who were infected with the coronavirus (12 patients). Their parents were asked to indicate what new or exacerbated symptoms their children exhibited during the course of the illness. The most commonly exacerbated symptom after confirmed COVID-19 infection was mood lability (58.33% of cases), followed by OCD, tics and sensory symptoms. This is important, given the hypothesis that additional infections may trigger symptoms of PANS/PANDAS [31].

COVID-19 infection can not only result in an exacerbation of the condition of children already affected by PANS but can also eventually trigger new-onset PANS. Indeed, two independent case series each reported two cases of young adolescents showing a temporal correlation between COVID-19 infection and PANSs onset [51,52]. In Efe [51], after asymptomatic SARS-CoV-2 infection, two sisters showed an abrupt onset of restricted foot intake two weeks after infection. During the active phase of the infection, they were both socially isolated for two weeks. Pavone et al. [52] reported the cases of two unrelated children diagnosed with PANS following the abrupt onset of PANS-related symptoms 2 weeks after a positive COVID-19 nasopharyngeal swab. The first child showed restricted food intake and OCD symptoms. The second experienced OCD symptoms and tic onset. 

The temporal association between the emergence of new cases of PANS and infection with SARS-Cov-2 may suggest a causative trigger role of COVID-19 in the development of central nervous system autoimmunity.

#### Cognitive Function Alterations

It is noteworthy that SARS-CoV-2 can also result in long-lasting symptoms related to cognitive functions. Indeed, a lack of concentration, for example, has been shown to be one of the most prevalent PACS symptoms in both adults and children [28,54]. No study has directly evaluated the impact of COVID-19 infection on PANS patients’ cognition. However, considering the findings showing alterations following infection in other pediatric samples, new studies should investigate this potential effect.

As reported previously, PANDAS reflects the selective involvement of cortico-striatal networks. Thus, patients with PANDAS may exhibit distinct neuropsychological profiles of vulnerability within cognitive, affective or behavioral domains [55,56,57]. Specifically, weakness in aspects of executive function related to cognitive efficiency may follow the disruption of basal ganglia networks, including inattention, cognitive slowing (slow processing speed), reduced verbal initiation, difficulties with set-shifting, disinhibition and a reduced working memory.

Patients with PANDAS had greater difficulty in sustaining attention, inhibiting responses to stimuli and quickly integrating and acting upon new information (i.e., they exhibited a slower processing speed) [58]. PACS patients showed executive function alterations post-COVID-19 [59], including alterations in attention [54]. In line with this finding, in the case reports of new-onset PANS following COVID-19, attention-deficit and decreased academic performance were observed [51,52].

Interestingly, alterations in cortico-striato-thalamo circuits in COVID-19 patients have also been related to cognitive symptoms [60]. Moreover, we found an association between executive function alterations and inflammatory markers in post-COVID-19 subjects (IL-6, fibrinogen). Given that PANS is characterized by the disruption of the striatal circuits and high levels of cytokines, including IL-6, the potential effects of SARS-CoV-2 in worsening inflammation and inducing immune responses should also be investigated in regard to the potential impacts of cognitive functions, considering that one of the main symptoms of COVID-19 is brain fog.

### 4.2. Vaccine Hesitancy and Vaccine-Related Neuroinflammation

Approximately 25% of caregivers of sampled PANS patients reported at the time of the study that they had no plans to vaccinate their children against COVID-19 [49]. This percentage is similar (28–33%) to those based on parental reports of vaccination refusal in other clinical populations [61]. Reluctance to vaccinate among PANDAS patients could also be due to the idea that people with autoimmune and inflammatory diseases might experience a worsening of their symptoms as a result of vaccination. An observational study comparing more than 1000 people with neuroinflammation diseases and 500 controls found no difference in the frequency of vaccine side effects and no evidence of an impact of vaccination on pre-existing symptoms [62]. However, a rare risk of autoimmune disorder following vaccination was also reported [63,64]. In any case, although rare, cases of neuropsychiatric symptoms following vaccination again highlight the importance of considering and assessing autoimmunity and inflammation markers in children in the COVID-19 age.

## 5. A Case Series

To further demonstrate the implications of SARS-CoV-2 infection for patients with PANDAS, here, we report five cases of adolescents admitted to the outpatient neuropsychiatric unit of the Institute of Neuroscience (Florence, Italy) between July and September 2022. The adolescents were previously diagnosed with PANDAS after a streptococcal infection. When they arrived at the institute, they had already been infected with SARS-CoV-2 and experienced (Cases 1 and 5) one month of isolation. All patients showed a worsening of tics and repetitive behaviors two weeks after having a negative PCR result. Moreover, they experienced increased social anxiety and depressive thoughts. The psychometric scale known as the Yale Global Tic Severity Scale was administered and showed a moderate impairment associated with tics. Interestingly, when observing the symptom checklist, higher percentages of both motor and vocal tics were reported as currently presented rather than ever-presented. The patients also complained of a lack of concentration. The Continuous Performance Test (CPT) and the Stop Signal Task showed impairments in attentional and executive functions. The laboratory test results are reported in Table 3.

In these patients, an exacerbation of PANS-related symptomatology occurred after SARS-CoV-2 infection, which was not related to TAS, anti-DNAsi B or streptococcus. In Cases 2–4, a reactivation of EBV was observed, which was associated with an increase in inflammatory markers (IL-6, fibrinogen). EBV could account for the exacerbation of PANDAS. In Cases 1–5, EBV was not reactivated. However, elevated levels of IL-6 were observed in both cases, while elevated fibrinogen and CRP were only observed in Case 1. In these two cases, considering the fact that the patients experienced a longer period of isolation during the active infection phase (one month), social distancing and isolation could have played a role in their complications of PANDAS symptoms. Indeed, inflammatory markers have been correlated with lockdown experiences [65]. Importantly, the exacerbation was not related to a direct effect of SARS-CoV-2. Moreover, regarding the cognitive alterations experienced by these five patients, PACS-related executive function alterations have been correlated with increased IL-6 levels [59], which were above the threshold for all the five of the patients. In the previous case reports described by Efe [51] and Pavone et al. [52], the onset of symptoms was not associated with a reactivation of EBV, although the authors reported an increase in inflammatory markers. Our case series, by showing that two possible mechanisms could account for the clinical picture, allow us to go a step further in our comprehension of the pathogenic mechanisms of PACS. The associations between behavioral reports and immunological findings are in favor of the hypothesis defining autoimmunity and EBV reactivation as potential trigger agents.

## 6. Possible Mechanisms: Inflammation and Epstein–Barr Virus Reactivation

The question of how COVID-19 may cause neurological and psychiatric symptoms in affected children is a debated issue. Lin and colleagues [66] advanced the hypothesis that COVID-19 causes an inflammatory and autoimmune response. Indeed, COVID-19 is correlated with aberrant and excessive inflammation [67]. As a result of the immune host’s antiviral response, neuropsychiatric abnormalities in COVID-19 patients have been correlated with greater levels of pro-inflammatory cytokines, such as IL-6, IL-2, IL-17 and TNF [68]. Peripheral cytokines may cause neuropsychiatric symptoms, even in the absence of SARS-CoV-2 infiltration into the CNS. In fact, they can trigger neuroinflammatory reactions and/or compromised blood–brain barrier integrity, which results in the migration of peripheral immune cells into the CNS, activation of the microglia and disruption of neurotransmission [42,68]. In particular, basal ganglia GABAergic transmission has been linked to changes caused by exposure to pro-inflammatory cytokines. The hypo-dopaminergic state of the basal ganglia is understood to be caused by other pro-inflammatory mediators, such interferon-alpha, which is believed to be a potential initiating factor for psychiatric disorders [69]. This is particularly relevant to PANS, considering the already disrupted functioning of the basal ganglia [44].

**Figure 1:**
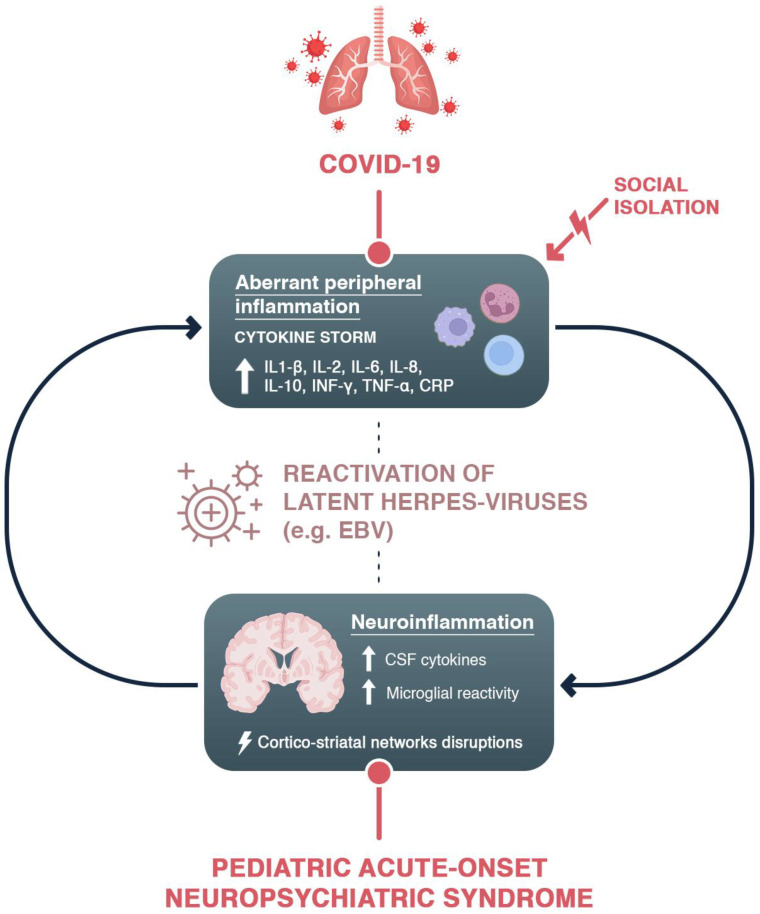
The hypothesized mechanism of the effects of COVID-19 infection on PANS. COVID-19, as well as social isolation related to the pandemic, could have triggered an immune response which led to a reactivation of silent viruses and to aberrant inflammation, which could target the cortico-striatal networks. Considering the involvement of these circuits in the symptomatology of PANS and further disruption caused by the phenomena elicited post-COVID, PANS patients could have experienced an exacerbation of their symptoms.

The immunological dyshomeostasis induced by a new virus is another pathophysiological mechanism that may play a role in PACS symptoms. The pathogenesis of long-term COVID-19 has been attributed to abnormal inflammatory responses, persistent SARS-CoV-2 reservoirs in particular tissues that cause post-infection morbidity, the reactivation of pathogens, host microbiome changes and molecular mimicry between SARS-CoV-2 and proteins [70]. SARS-CoV-2 may stimulate an abnormal immune response that results in autoimmunity later on, with secondary nervous system damage in susceptible people (particularly those with allergies or a history of autoimmune illnesses, as occurs in PANS). Prior to the onset of autoimmunity, viral infections may cause an inflammatory environment that supports and encourages the “molecular mimicry” phenomenon by increasing the level of host antibodies or lymphocytes that are cross-reactive with both viral antigens and self-antigens [19]. In line with this, in children with neuropsychiatric onset after COVID-19, increased levels of lymphocytes have frequently been found [29,51]. Considering this evidence, we suppose that the etiology of neuropsychiatric complications in COVID-19 could be multifactorial (Figure 1), as a result of a complex interplay between systemic and brain inflammation and environmental stress in vulnerable individuals.

Concerning the relationship between SARS-CoV-2 and PANS, it is necessary to distinguish between the mechanism that leads to the new onset of PANS after COVID-19 infection and the exacerbation of symptoms in PANS patients following infection. In the first case, it can be hypothesized that SARS-CoV-2 acts as a causal agent of the new onset of PANS or reactivates EBV, triggering PANS. Indeed, as reported previously, EBV may be the trigger of PANS. Moreover, SARS-CoV-2 can also result in the activation of other viruses, specifically herpes viruses. For a detailed list of potential triggers, see Table 2. In the second case, the mechanism exposed above could be the cause. Therefore, systemic inflammation in response to SARS-CoV-2 could exacerbate neuropsychiatric symptoms. As COVID-19 triggers systemic inflammation, it may also activate the microglia, lower the levels of monoamines and trophic factors, increase the levels of glutamate and N-methyl-d-aspartate and cause excitotoxicity [71]. Overall, these series of events may cause pre-existing neuropsychiatric problems to worsen [71]. Therefore, the pathogenesis of the concomitant neuropsychiatric symptoms may include neuro-inflammatory pathways [72]. The exacerbation of symptoms may be related to inflammatory responses to SARS-CoV-2 virus that affect PANS symptoms. The immunological profiles of PANS patients have also shown some non-univocal alterations [46]. Pro-inflammatory chemokines, as well as CRP, were significantly elevated in pediatric patients with neuropsychiatric disorders and COVID-19 compared to controls [73]. Moreover, children with COVID-19 and neuropsychiatric complications may respond to immunotherapy. These data highlight the possibility of a CNS–immune-related mechanism in children with COVID-19 who experience neuropsychiatric symptoms [74]. 

In cases where symptoms persist for months after the acute phase of COVID-19 and after negativization, the reactivation of other viruses, such as EBV, can cause an immune response leading to the persistence of the symptomatology. Indeed, herpes viruses are neutropic and neurovirulent; thus, they can infect nervous cells and produce neuropsychiatric symptoms [75]. Gold et al. [76] found that 66.7% of Long COVID subjects were positive for Epstein–Barr virus (EBV) reactivation. Moreover, Klein et al. [77] found that Long COVID individuals had higher titers of anti-EBV antibodies. In these cases, the symptoms did not decrease until the reactivated viruses and the associated immune responses were adopted as the target of the treatment. In accordance with this hypothesis, an observational study showed that in Long COVID patients, the inflammatory profile was correlated with alterations in executive functions, with potential effects of EBV reactivation [59]. Together, these studies make a strong case for the argument that Long COVID chronic inflammation may be due, in part, to a reactivated virus such as EBV. The question of whether this exacerbation of PANS symptoms is due to SARS-CoV-2 with a reactivated herpes virus needs to be considered. This pathogenic mechanism can explain two of the cases reported here.

Additionally, the pandemic’s impact on people who were not physically affected by the virus may be explained, again, by the inflammatory theory. In fact, many people who are subjected to lockdowns and social isolation are affected by loneliness-related raised levels of inflammatory indicators [78,79]. Koyama et al. [79] found that people who were socially isolated and felt lonely had higher NLR. Interestingly, people who were not socially isolated but felt lonely had lower CRP. In the study of Brusaferri and colleagues [65] inflammatory markers such as IL-6 were also shown to be increased after the lockdown in healthy individuals. A metanalysis showed loneliness and social isolation are associated with inflammatory markers such as IL-6, CRP and fibrinogen [80]. In conclusion, being socially isolated and feeling lonely have both been associated with chronic inflammation. As a consequence, neuroimmune activation may be a possible mechanism underlying many of the symptoms experienced by uninfected individuals during the pandemic. This pathogenic mechanism could account for the exacerbation of PANDAS symptoms in the other cases reported here, in which increased levels of IL-6 were observed, together with CRP and fibrinogen alterations.

In conclusion, herein, COVID-related viral infection was considered as a trigger of autoimmune phenomena, together with comorbid autoimmune disorders (PANS), age and one’s surrounding environment. In this sense, the psychological distress caused by the environmental factors that the pandemic forced upon all of us could have worked jointly to worsen the autoimmune phenomena triggered by COVID-19 beyond what occurs in cases of seasonal influenza. This indicates the necessity of uncovering the involved mechanisms and defining new operational clinical interventions.

## 7. Conclusions and Perspectives

In this review, we exposed the impact of COVID-19 on children with a previous diagnosis of PANS, as well its potential impact on new-onset PANS. Previous studies showing the effects of COVID-19 on PANS did not expose any potential mechanism of action and did not consider the exacerbation and new onset of PANDAS cases together. They considered only the consequences of the pandemic, without considering the impacts of the illness and the virus on the brain. Conversely, herein, we proposed a potential mechanism involving autoimmunity and EBV reactivation as potential trigger agents. Furthermore, we presented five cases of adolescents with PANDAS whose symptomatology increased following acute SARS-CoV-2 infection. Three cases showed a reactivation of EBV, as well as increased inflammatory markers. In the other two cases, EBV was not reactivated, but the inflammatory panel was altered. Different mechanisms could account for the cases reported, but this question needs to be investigated further. However, what is certain is that it is important for healthcare professionals to be aware that COVID-19 infection has the potential to exacerbate PANS/PANDAS symptoms in this population or even act as a factor triggering new onset in certain cases. The symptoms of PANS/PANDAS may be intensified even in children who have not had COVID-19 because of causes or stresses related to the pandemic. The pandemic may have had a detrimental influence on general social and emotional functioning. Thus, support methods and therapies should be suggested to address these complex problems.

Intravenous immunoglobulin (IVIG) has shown clinical efficacy in critical ill patients with COVID-19 [81]. This treatment is also prescribed to PANDAS patients [82], suggesting, again, a potential relationship between the underlying immune profiles of PANS and COVID-19 patients. Moreover, SSRIs, another treatment commonly prescribed to PANDAS patients, could be a protective factor in COVID-19 infections [83]. Therefore, it is also possible that PANDAS patients are protected by their treatments. No studies have addressed this point. We hope that future studies will investigate the neuroinflammatory profiles of PANS patients who have been infected with the coronavirus and will compare the incidence and symptom severity between those who were treated with SSRIs and those treated differently. Again, this highlights that a neuroscience-based approach designed to reduce SARS-CoV-2-related mental health sequelae is needed [3].

## Figures and Tables

**Table 1:** Pediatric Acute-Onset Neuropsychiatric Disorders (PANS) diagnostic criteria. Modified from Swedo et al. [32].

*Criteria*
Abrupt onset of OCD or restricted food intake
2. Presence of at least 2 of the following symptoms: Anxiety Emotional lability and/or depression Irritability, aggression Behavioral regression Worsening of school performance Sensory or motor abnormalities Sleep disturbances, enuresis or urinary frequency
3. Symptoms not better explained by another disorder, such as Sydenham chorea, Tourette disorder or others.

**Table 2:** Studies investigating PANS/PANDAS and COVID-19.

Reference	Study Type	Sample	Notes
O’Dor et al., 2022 [49]	Survey	254 minors	Self-reported (by caregivers) worsening of symptoms during the pandemic
Guido et al., 2021 [50]	Survey	108 minors	Self-reported (by caregivers) worsening of symptoms during the pandemic
Efe, 2022 [51]	Case series	2 adolescents	Onset of PANS temporarily associated with COVID-19 infection
Pavone et al., 2021 [52]	Case series	2 adolescents	Onset of PANS temporarily associated with COVID-19 infection

**Table 3:** Lab test results of the five cases.

*Lab Test*	Lab Ref Value	Case 1	Case 2	Case 3	Case 4	Case 5
*EBV anticorpi (VCA IgG)* (U/mL)	<20	12	27	42	38	15
*IL-6* (pg/mL)	<4.4	8.2	5.6	7.8	12.8	5.2
*VES* mm/h	2–28	12	24	23	18	5
*TAS* Ul/mL	<250	112	211	156	167	198
*CRP* mg/dL *<1.00*	<1.00	1.5	<1.00	1.8	<1.00	<1.00
*Anticorpi anti-DNASI-B* (U/mL)	<200	127	182	98	45	131
*Fibrinogen* (mg/dL)	180–400	421	736	531	386	350
*D-dimer* (µg/L)	<500	410	118	257	511	689

## Data Availability

Not applicable.
